# Crystal structure of ebastinium 3,5-di­nitro­benzoate

**DOI:** 10.1107/S205698901701324X

**Published:** 2017-09-19

**Authors:** Mohammed A. E. Shaibah, Belakavadi K. Sagar, Hemmige S. Yathirajan, S. Madan Kumar, Christopher Glidewell

**Affiliations:** a Department of Studies in Chemistry, University of Mysore, Manasagangotri, Mysuru-570 006, India; b Department of Studies in Chemistry, Mangalore University, Mangalagangotri-574 199, India; c School of Chemistry, University of St Andrews, St Andrews, Fife KY16 9ST, UK

**Keywords:** mol­ecular structure, disorder, conformation, hydrogen bonding, supra­molecular assembly, crystal structure

## Abstract

Ebastine, 4-(benzhydr­yloxy)-1-[4-(4-*tert*-butyl­phen­yl)-4-oxobut­yl]piperidine, reacts with 3,5-di­nitro­benzoic acid in methanol solution to give the title 1:1 salt, ebastinium 3,5-di­nitro­benzoate, C_32_H_40_NO_2_^+^·C_7_H_3_N_2_O_6_^−^. In the cation, the disubstituted aryl ring exhibits orientational disorder over two sets of atomic sites having occupancies 0.706 (4) and 0.294 (6), with a dihedral angle of 41.2 (5)° between the two orientations: the bulky Ph_2_CH—O– substituent occupies an axial site on the piperidine ring. The two ions in the selected asymmetric unit are linked by a nearly linear N—H⋯O hydrogen bond and this, in combination with two C—H⋯O hydrogen bonds, links the ions into complex sheets.

## Abstract 2

In the cation of the title mol­ecular salt, one of the non-H substituents on the piperidine ring occupies an equatorial site and the other an axial site. The ions are linked into sheets by a combination of one N—H⋯O and two C—H⋯O hydrogen bonds.

## Chemical context   

Ebastine, or 4-(benzhydr­yloxy)-1-[4-(4-*tert*-butyl­phen­yl)-4-oxo­but­yl]piperidine, is a non-sedating second generation H_1_ receptor antagonist, which is effective in the treatment of both allergic rhinitis, whether seasonal or perennial, and chronic idiopathic urticaria (Wiseman & Faulds, 1996 ▸; Van Cauwenberge *et al.*, 2004 ▸). The structure of ebastine has been the subject of two recent reports (Cheng *et al.*, 2005 ▸: Sharma *et al.*, 2015 ▸). Herein, we report the mol­ecular and supra­molecular structure of the 1:1 salt ebastinium 3,5-di­nitro­benzoate (I), formed in the reaction between ebastine and 3,5-di­nitro­benzoic acid.
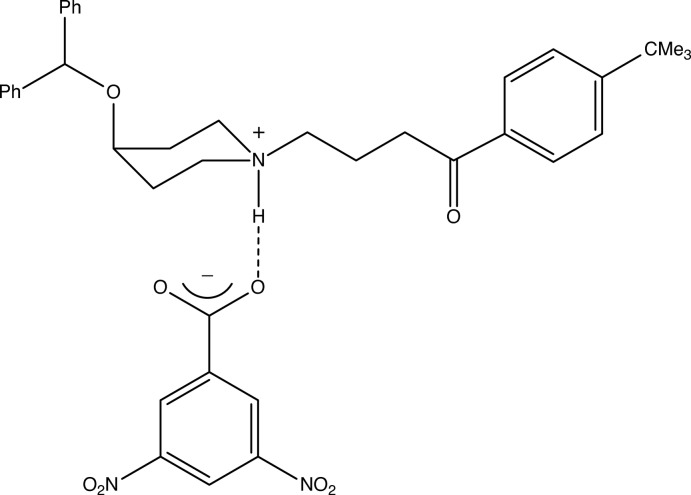


## Structural commentary   

**Figure 1:**
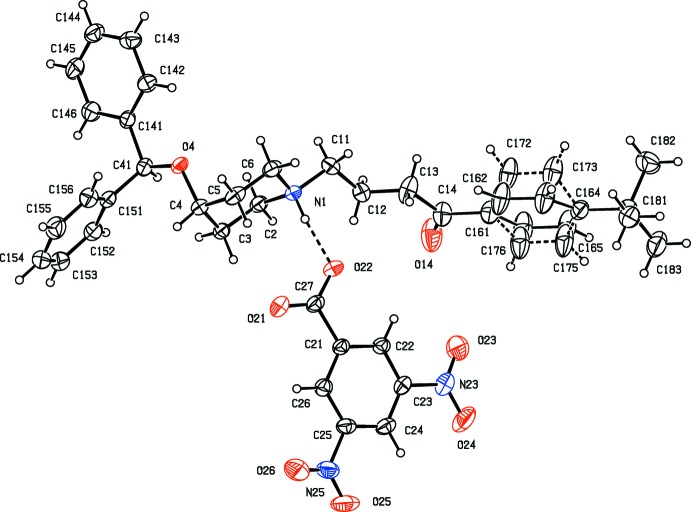
The mol­ecular structure of the ionic components of compound (I), showing the atom-labelling scheme, the N—H⋯O hydrogen bond within the selected asymmetric unit, and the orientational disorder of the disubstituted aryl ring (the major component is drawn with full lines and the minor component with broken lines). Displacement ellipsoids are drawn at the 30% probability level and, for clarity, a few of the atom labels have been omitted.

The title compound (I), consists of an N-protonated ebastinium cation and a 3,5-di­nitro­benzoate anion (Fig. 1 ▸), which are linked within the selected asymmetric unit a by a fairly short and nearly linear N—H⋯O hydrogen bond (Fig. 1 ▸, Table 1 ▸). The disubstituted aryl ring in the cation is disordered over two sets of atomic sites having occupancies 0.706 (4) for the major ring orientation, labelled C161–C166, and 0.294 (4) for the minor orientation, labeled C171–C176: the dihedral angle between these two ring planes is 41.2 (5)° (Fig. 1 ▸). The piperidine ring adopts an almost perfect chair conformation, with a ring-puckering angle, calculated for the atom sequence (N1,C2,C3,C4,C5,C6) of θ = 0.0 (3)°, identical within experimental uncertainty to the idealized value for a perfect chair form of θ = 0.0° (Boeyens, 1978 ▸). However, although the non-H substituent at atom N1 in the ring occupies an equatorial site, as expected, the bulky Ph_2_CHO substituent at atom C4 unexpectedly occupies an axial site. This observation is the more surprising since in ebastine itself, both non-H substit­uents on the piperidine ring occupy equatorial sites (Cheng *et al.*, 2005 ▸: Sharma *et al.*, 2015 ▸). The 3,5-di­nitro­benzoate anion in compound (I) is nearly planar: the dihedral angles between the aryl ring and the substituents at atoms C21, C23 and C25 are 1.4 (2), 4.2 (2) and 10.7 (2)°, respectively: only the O atoms of the 5-nitro group are significantly displaced from the mean plane of the anion as a whole, 0.219 (2) Å for atom O25 and 0.187 (2) Å for atom O26: the r.m.s. deviation from the mean plane for the entire anion is only 0.082 Å.

## Supra­molecular features   

**Figure 2:**
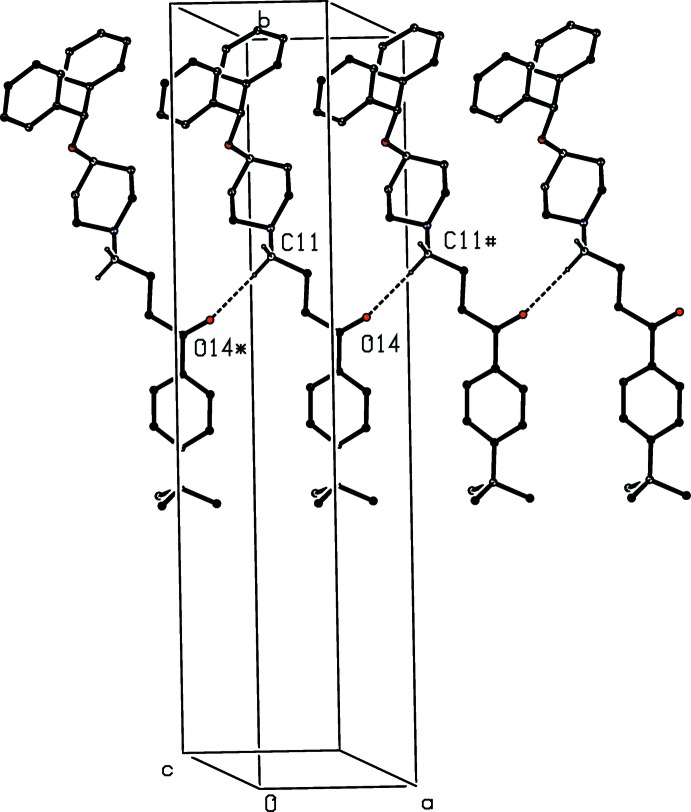
Part of the crystal structure of compound (I), showing a hydrogen-bonded *C*(6) chain of cations running parallel to [100]. For clarity, the anions, the minor disorder component of the cation, and the H atoms bonded to carrier atoms not involved in the motif shown have been omitted. The atoms marked with an asterisk (*) or a hash (#) are at the symmetry positions (−1 + *x*, *y*, *z*) and (1 + *x*, *y*, *z*) respectively.

**Figure 3:**
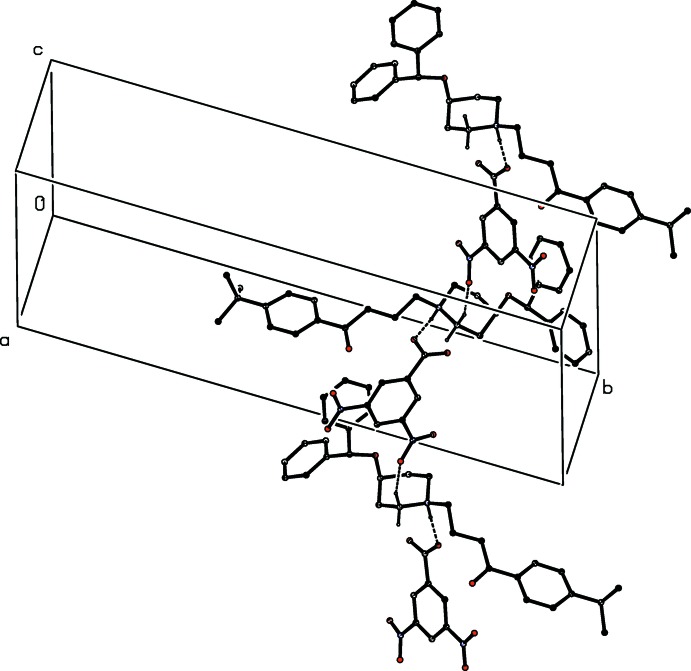
Part of the crystal structure of compound (I), showing a hydrogen-bonded 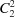
(11) chain running parallel to [20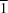]. For clarity, the minor disorder component of the cation, and the H atoms bonded to C atoms not involved in the motif shown have been omitted.

In addition to the N—H⋯O hydrogen bond within the selected asymmetric unit, already noted (*cf*. Fig. 1 ▸ and Table 1 ▸), there are two C—H⋯O hydrogen bonds in the crystal of compound (I), which link the components into complex sheets, whose formation can, however, be readily analysed in terms of two simple, one-dimensional sub-structures (Ferguson *et al.*, 1998*a* ▸,*b* ▸; Gregson *et al.*, 2000 ▸). In the simpler of the two sub-structures, cations related by translation are linked by a single C—H⋯O hydrogen bond to form a *C*(6) chain running parallel to the [100] direction (Fig. 2 ▸, Table 1 ▸). The second sub-structure involves the cations and the anions, and a combination of the N—H⋯O hydrogen bond and a second C—H⋯O hydrogen bond links ions related by a *c*-glide plane into a 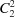
(11) chain, running parallel to the [20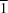] direction, in which cations and anions alternate (Fig. 3 ▸, Table 1 ▸). The combination of these two chain motifs generates a sheet lying parallel to (010) in the domain 0.5 < *y* < 1.0, and a second such sheet, related to the first by inversion, lies in the domain 0.0 < *y* < 0.5, but there are no direction-specific inter­actions between adjacent sheets. It is inter­esting to note that none of the hydrogen bonds in compound (I) involves the Ph_2_CHO substituent, so that direction-specific inter­actions cannot be held responsible for the location of this substituent at an axial site on the piperidine ring.

## Database survey   

**Figure 4:**
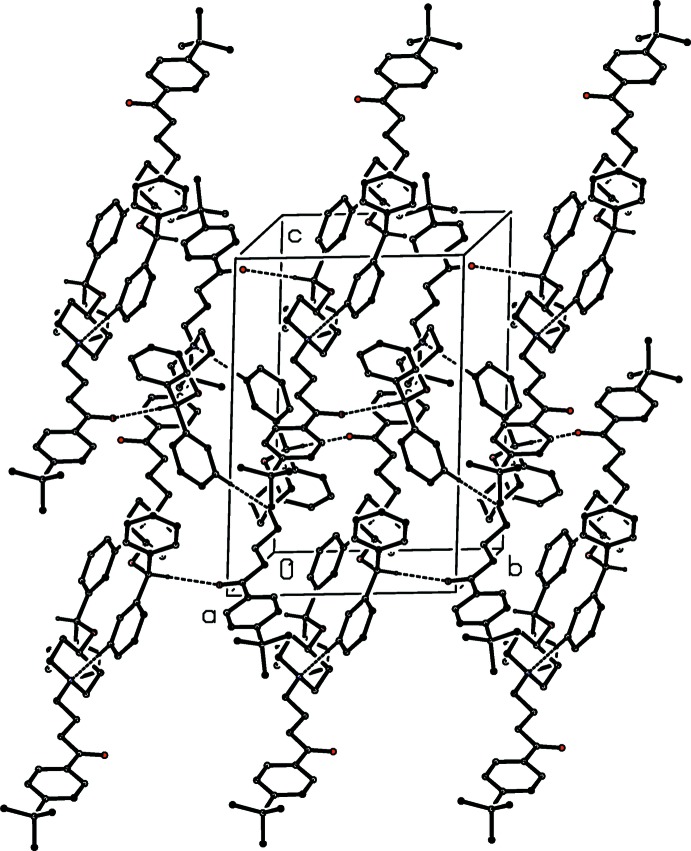
Part of the crystal structure of ebastine showing the formation of a hydrogen-bonded sheet of 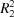
(20) and 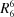
(48) rings. The original atomic coordinates (Sharma *et al.*, 2015 ▸) have been used and, for the sake of clarity, the H atoms not involved in the motifs shown have been omitted.

The mol­ecular structure of neutral ebastine (Cheng *et al.*, 2005 ▸; Sharma *et al.*, 2015 ▸) differs from that of the ebastinium cation in compound (I) in two significant respects. Firstly, there is no disorder in the neutral compound as opposed to the orientation disorder of the disubstituted aryl ring in (I) and secondly, both of the non-H substituents on the piperidine ring occupy equatorial sites in the neutral compound as opposed to the presence of one axial and one equatorial substituent in (I). Neither of the two reports on the structure of ebastine gave any description of the supra­molecular assembly: one (Cheng *et al.*, 2005 ▸) noted the presence of hydrogen bonds, but the second (Sharma *et al.*, 2015 ▸) did not record these. Accordingly, we have now examined the supra­molecular assembly of ebastine using the most recently reported atomic coordinates (Sharma *et al.*, 2015 ▸): a combination of one C—H⋯N hydrogen bond and one C—H⋯O hydrogen bond links the mol­ecules into sheets lying parallel to (100) and containing 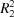
(20) and 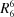
(48) rings, both centrosymmetric, arranges in chess board fashion (Fig. 4 ▸). Structures have also been reported recently for some structurally related compounds with pharmacological activity, including the picrate salt of the anti­cholinergic drug propiverine, 4-(2,2-diphenyl-2-prop­oxy­acet­oxy)-1-meth­ylpiperidin-1-ium picrate (Jasinski *et al.*, 2009 ▸), and the anti-spasmodic drug pargeverine, *N*,*N*-dimeth­yl-[2-(2,2-diphen­yl)-2-prop-2-yn­yloxy)acet­oxy]ethyl­amine and its picrate and (2*R*,3*R*)-(hydrogentartrate) salts (Shaibah *et al.*, 2017 ▸).

## Synthesis and crystallization   

A sample of ebastine was a gift from RL Fine Chem, Pvt. Ltd., Bengaluru, India. For the synthesis of compound (I), ebastine (100 mg, 0.20 mmol) and 3,5-di­nitro­benzoic acid (45 mg, 0.20 mmol) were dissolved in hot methanol and held at 333 K for 30 min, with magnetic stirring throughout. The resulting solution was then allowed to cool slowly to room temperature, giving colourless block-like crystals (m.p. 424–428 K).

## Refinement   

Crystal data, data collection and structure refinement details are summarized in Table 2 ▸. Three low-angle reflections (021), (002) and (012), which had been attenuated by the beam stop, were omitted from the refinements. It was apparent from an early stage in the refinement that the disubstituted aryl ring was disordered over two sets of atomic sights having unequal occupancies, and corresponding to different orientations of this ring relative to its substituents. For the minor orientation, the bonded distances and the 1,3-non-bonded distances were restrained to be the same as the corresponding distances in the major orientation, subject to s.u.s of 0.01 and 0.02 Å, respect­ively: in addition, the anisotropic displacement parameters for corresponding pairs of atomic sites were constrained to be equal. All H atoms, other than those in the minor disorder components, were located in difference-Fourier maps. The C-bound H atoms were all treated as riding atoms in geometrically idealized positions: C—H 0.93 Å (aromatic), 0.96 Å (CH_3_), 0.97 Å (CH_2_) or 0.98 Å (aliphatic C—H), with *U*_iso_(H) = 1.5*U*_eq_(C-meth­yl) and 1.2*U*_eq_(C) for other H atoms. The methyl groups were permitted to rotate but not to tilt. For the H atom bonded to the N atom, the atomic coordinates were refined with *U*_iso_(H) = 1.2*U*_eq_(N), giving an N—H distance of 0.99 (3) Å. Subject to these conditions, the occupancies of the two disordered components refined to 0.706 (4) and 0.294 (4). In the final analysis of variance there was a large value, 15.256, of *K* = [mean(*F*_o_^2^)/mean(*F*_c_^2^)] for the group of 867 very weak reflections having *F*_c_/*F*_c_(max) in the range 0.000 < *F*_c_/*F*_c_(max) < 0.005.

## Supplementary Material

Crystal structure: contains datablock(s) global, I. DOI: 10.1107/S205698901701324X/su5391sup1.cif

Structure factors: contains datablock(s) I. DOI: 10.1107/S205698901701324X/su5391Isup2.hkl

Supporting information file. DOI: 10.1107/S205698901701324X/su5391Isup3.cml

CCDC reference: 1574718

Additional supporting information:  crystallographic information; 3D view; checkCIF report

## Figures and Tables

**Table 1:** Hydrogen-bond geometry (Å, °)

*D*—H⋯*A*	*D*—H	H⋯*A*	*D*⋯*A*	*D*—H⋯*A*
N1—H1⋯O22	0.99 (3)	1.66 (2)	2.634 (3)	167 (2)
C2—H2*A*⋯O25^i^	0.97	2.50	3.444 (3)	163
C11—H11*A*⋯O14^ii^	0.97	2.49	3.358 (4)	150

Symmetry codes: (i) 
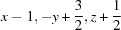
; (ii) 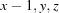.

**Table 2:** Experimental details

Crystal data	
Chemical formula	C_32_H_40_NO_2_·C_7_H_3_N_2_O_6_
*M*_r_	681.76
Crystal system, space group	Monoclinic, *P*2_1_/*c*
Temperature (K)	293
*a*, *b*, *c* (Å)	5.9168 (3), 28.3733 (12), 21.0782 (11)
β (°)	97.836 (5)
*V* (Å^3^)	3505.6 (3)
*Z*	4
Radiation type	Mo *K*α
μ (mm^−1^)	0.09
Crystal size (mm)	0.23 × 0.21 × 0.18

Data collection	
Diffractometer	Rigaku Saturn724
Absorption correction	Multi-scan (*SADABS*; Krause *et al.*, 2015 ▸)
*T*_min_, *T*_max_	0.956, 0.984
No. of measured, independent and observed [*I* > 2σ(*I*)] reflections	40112, 7331, 4388
*R*_int_	0.061
(sin θ/λ)_max_ (Å^−1^)	0.629

Refinement	
*R*[*F* ^2^ > 2σ(*F* ^2^)], *wR*(*F* ^2^), *S*	0.065, 0.179, 1.05
No. of reflections	7331
No. of parameters	470
No. of restraints	22
H-atom treatment	H atoms treated by a mixture of independent and constrained refinement
Δρ_max_, Δρ_min_ (e Å^−3^)	0.20, −0.25

Computer programs: *CrystalClear* (Rigaku, 2011 ▸), *SHELXS86* (Sheldrick, 2008 ▸), *SHELXL2014* (Sheldrick, 2015 ▸) and *PLATON* (Spek, 2009 ▸).

## supplementary crystallographic information

### Crystal data

**Table:**

C_32_H_40_NO_2_^+^·C_7_H_3_N_2_O_6_^−^	*F*(000) = 1448
*M**_r_* = 681.76	*D*_x_ = 1.292 Mg m^−^^3^
Monoclinic, *P*2_1_/*c*	Mo *K*α radiation, λ = 0.71073 Å
*a* = 5.9168 (3) Å	Cell parameters from 10431 reflections
*b* = 28.3733 (12) Å	θ = 2.4–31.2°
*c* = 21.0782 (11) Å	µ = 0.09 mm^−^^1^
β = 97.836 (5)°	*T* = 293 K
*V* = 3505.6 (3) Å^3^	Block, colourless
*Z* = 4	0.23 × 0.21 × 0.18 mm

### Data collection

**Table:**

Rigaku Saturn724 diffractometer	4388 reflections with *I* > 2σ(*I*)
Radiation source: fine focus sealed tube	*R*_int_ = 0.061
φ and ω scans	θ_max_ = 26.6°, θ_min_ = 2.4°
Absorption correction: multi-scan (SADABS; Krause *et al.*, 2015)	*h* = −7→7
*T*_min_ = 0.956, *T*_max_ = 0.984	*k* = −35→35
40112 measured reflections	*l* = −26→25
7331 independent reflections	

### Refinement

**Table:**

Refinement on *F*^2^	Primary atom site location: structure-invariant direct methods
Least-squares matrix: full	Secondary atom site location: difference Fourier map
*R*[*F*^2^ > 2σ(*F*^2^)] = 0.065	Hydrogen site location: mixed
*wR*(*F*^2^) = 0.179	H atoms treated by a mixture of independent and constrained refinement
*S* = 1.05	*w* = 1/[σ^2^(*F*_o_^2^) + (0.0661*P*)^2^ + 1.0412*P*] where *P* = (*F*_o_^2^ + 2*F*_c_^2^)/3
7331 reflections	(Δ/σ)_max_ = 0.001
470 parameters	Δρ_max_ = 0.20 e Å^−^^3^
22 restraints	Δρ_min_ = −0.25 e Å^−^^3^

### Special details

**Table:**

Geometry. All esds (except the esd in the dihedral angle between two l.s. planes) are estimated using the full covariance matrix. The cell esds are taken into account individually in the estimation of esds in distances, angles and torsion angles; correlations between esds in cell parameters are only used when they are defined by crystal symmetry. An approximate (isotropic) treatment of cell esds is used for estimating esds involving l.s. planes.

### Fractional atomic coordinates and isotropic or equivalent isotropic displacement parameters (Å^2^)

**Table:**

	*x*	*y*	*z*	*U*_iso_*/*U*_eq_	Occ. (<1)
N1	0.3135 (4)	0.72898 (6)	0.37455 (11)	0.0510 (5)	
H1	0.374 (4)	0.7192 (8)	0.3349 (12)	0.061*	
C2	0.4460 (4)	0.77114 (8)	0.40021 (11)	0.0479 (6)	
H2B	0.6062	0.7629	0.4097	0.057*	
H2A	0.3938	0.7811	0.4398	0.057*	
C3	0.4185 (4)	0.81101 (8)	0.35285 (11)	0.0457 (6)	
H3A	0.4826	0.8018	0.3147	0.055*	
H3B	0.5027	0.8382	0.3713	0.055*	
C4	0.1701 (4)	0.82448 (8)	0.33437 (11)	0.0457 (6)	
H4	0.1579	0.8488	0.3011	0.055*	
C5	0.0386 (4)	0.78137 (9)	0.30906 (12)	0.0553 (7)	
H5A	−0.1219	0.7892	0.2994	0.066*	
H5B	0.0916	0.7713	0.2697	0.066*	
C6	0.0676 (4)	0.74172 (9)	0.35682 (13)	0.0575 (7)	
H6A	0.0043	0.7510	0.3950	0.069*	
H6B	−0.0160	0.7144	0.3388	0.069*	
O4	0.0623 (3)	0.84055 (5)	0.38735 (7)	0.0484 (4)	
C41	0.1566 (4)	0.88157 (8)	0.41962 (11)	0.0460 (6)	
H41	0.3107	0.8739	0.4403	0.055*	
C141	0.0098 (4)	0.89282 (8)	0.47183 (11)	0.0453 (6)	
C142	−0.1660 (4)	0.86412 (9)	0.48464 (11)	0.0511 (6)	
H142	−0.1975	0.8367	0.4610	0.061*	
C143	−0.2974 (5)	0.87546 (10)	0.53237 (12)	0.0613 (7)	
H143	−0.4159	0.8557	0.5403	0.074*	
C144	−0.2527 (6)	0.91565 (10)	0.56772 (13)	0.0686 (8)	
H144	−0.3422	0.9236	0.5991	0.082*	
C145	−0.0755 (6)	0.94408 (10)	0.55665 (15)	0.0798 (10)	
H145	−0.0426	0.9711	0.5812	0.096*	
C146	0.0550 (6)	0.93287 (9)	0.50904 (14)	0.0719 (8)	
H146	0.1749	0.9525	0.5019	0.086*	
C151	0.1735 (4)	0.92244 (8)	0.37464 (11)	0.0469 (6)	
C152	0.3761 (5)	0.94689 (9)	0.37596 (14)	0.0612 (7)	
H152	0.5016	0.9380	0.4050	0.073*	
C153	0.3962 (6)	0.98416 (10)	0.33510 (18)	0.0770 (9)	
H153	0.5338	1.0003	0.3368	0.092*	
C154	0.2134 (7)	0.99730 (11)	0.29230 (17)	0.0808 (10)	
H154	0.2264	1.0222	0.2644	0.097*	
C155	0.0086 (6)	0.97356 (11)	0.29040 (15)	0.0754 (9)	
H155	−0.1161	0.9827	0.2613	0.091*	
C156	−0.0118 (5)	0.93637 (9)	0.33152 (13)	0.0603 (7)	
H156	−0.1504	0.9207	0.3302	0.072*	
C11	0.3376 (6)	0.68892 (10)	0.42095 (17)	0.0865 (11)	
H11A	0.2213	0.6656	0.4071	0.104*	
H11B	0.3101	0.7005	0.4625	0.104*	
C12	0.5687 (5)	0.66527 (9)	0.42801 (16)	0.0724 (9)	
H12A	0.6523	0.6664	0.4737	0.087*	
H12B	0.6523	0.6776	0.4014	0.087*	
C13	0.5462 (5)	0.61430 (10)	0.41343 (17)	0.0833 (10)	
H13A	0.4697	0.6108	0.3700	0.100*	
H13B	0.4481	0.6005	0.4417	0.100*	
C14	0.7637 (6)	0.58644 (10)	0.41954 (15)	0.0709 (8)	
O14	0.9451 (4)	0.60527 (8)	0.43408 (16)	0.1170 (10)	
C161	0.7488 (5)	0.53513 (9)	0.40590 (14)	0.0659 (8)	0.706 (4)
C162	0.5497 (7)	0.51590 (14)	0.3727 (3)	0.0902 (17)	0.706 (4)
H162	0.4245	0.5351	0.3599	0.108*	0.706 (4)
C163	0.5386 (7)	0.46828 (14)	0.3589 (3)	0.0907 (18)	0.706 (4)
H163	0.4054	0.4561	0.3364	0.109*	0.706 (4)
C164	0.7209 (5)	0.43792 (9)	0.37782 (13)	0.0587 (7)	0.706 (4)
C165	0.9157 (9)	0.45915 (18)	0.4048 (5)	0.103 (4)	0.706 (4)
H165	1.0458	0.4407	0.4147	0.124*	0.706 (4)
C166	0.9308 (9)	0.50664 (17)	0.4185 (4)	0.099 (3)	0.706 (4)
H166	1.0697	0.5192	0.4367	0.119*	0.706 (4)
C171	0.7488 (5)	0.53513 (9)	0.40590 (14)	0.0659 (8)	0.294 (4)
C172	0.5662 (15)	0.5073 (3)	0.4211 (5)	0.0902 (17)	0.294 (4)
H172	0.4497	0.5216	0.4396	0.108*	0.294 (4)
C173	0.5554 (15)	0.4594 (3)	0.4093 (5)	0.0907 (18)	0.294 (4)
H173	0.4382	0.4415	0.4223	0.109*	0.294 (4)
C174	0.7209 (5)	0.43792 (9)	0.37782 (13)	0.0587 (7)	0.294 (4)
C175	0.9160 (17)	0.4626 (4)	0.3793 (15)	0.103 (4)	0.294 (4)
H175	1.0483	0.4468	0.3726	0.124*	0.294 (4)
C176	0.9267 (18)	0.5104 (4)	0.3905 (12)	0.099 (3)	0.294 (4)
H176	1.0625	0.5262	0.3872	0.119*	0.294 (4)
C181	0.7009 (5)	0.38550 (9)	0.36135 (13)	0.0591 (7)	
C182	0.6091 (6)	0.35909 (12)	0.41549 (16)	0.0925 (11)	
H18A	0.5908	0.3264	0.4044	0.139*	
H18B	0.4643	0.3721	0.4220	0.139*	
H18C	0.7145	0.3622	0.4541	0.139*	
C183	0.9278 (5)	0.36413 (11)	0.35081 (18)	0.0903 (11)	
H18D	1.0326	0.3661	0.3897	0.135*	
H18E	0.9883	0.3811	0.3175	0.135*	
H18F	0.9059	0.3317	0.3386	0.135*	
C184	0.5349 (5)	0.37825 (11)	0.29982 (14)	0.0753 (8)	
H18G	0.5895	0.3949	0.2653	0.113*	
H18H	0.3872	0.3900	0.3058	0.113*	
H18I	0.5240	0.3453	0.2898	0.113*	
C21	0.7522 (4)	0.69940 (8)	0.19856 (11)	0.0431 (5)	
C22	0.8763 (4)	0.66079 (8)	0.22368 (11)	0.0458 (6)	
H22	0.8338	0.6448	0.2587	0.055*	
C23	1.0631 (4)	0.64614 (8)	0.19644 (12)	0.0486 (6)	
C24	1.1306 (4)	0.66812 (9)	0.14419 (12)	0.0525 (6)	
H24	1.2571	0.6579	0.1263	0.063*	
C25	1.0032 (4)	0.70587 (9)	0.11964 (11)	0.0505 (6)	
C26	0.8156 (4)	0.72192 (8)	0.14567 (11)	0.0493 (6)	
H26	0.7327	0.7476	0.1278	0.059*	
C27	0.5535 (4)	0.71822 (9)	0.23013 (13)	0.0504 (6)	
O21	0.4514 (3)	0.75292 (7)	0.20644 (9)	0.0689 (5)	
O22	0.5158 (3)	0.69618 (6)	0.27979 (9)	0.0649 (5)	
N23	1.1983 (4)	0.60593 (8)	0.22480 (13)	0.0672 (6)	
O23	1.1460 (4)	0.58894 (8)	0.27369 (12)	0.0920 (7)	
O24	1.3561 (4)	0.59235 (8)	0.19822 (12)	0.1010 (8)	
N25	1.0724 (5)	0.72964 (9)	0.06325 (12)	0.0700 (7)	
O25	1.2546 (4)	0.71894 (9)	0.04679 (11)	0.0974 (8)	
O26	0.9409 (5)	0.75808 (9)	0.03539 (11)	0.1034 (8)	

### Atomic displacement parameters (Å^2^)

**Table:**

	*U*^11^	*U*^22^	*U*^33^	*U*^12^	*U*^13^	*U*^23^
N1	0.0585 (13)	0.0405 (10)	0.0606 (13)	0.0002 (9)	0.0321 (11)	−0.0031 (10)
C2	0.0490 (14)	0.0496 (13)	0.0474 (14)	0.0035 (11)	0.0147 (11)	−0.0072 (11)
C3	0.0470 (14)	0.0421 (12)	0.0508 (14)	−0.0017 (10)	0.0168 (11)	−0.0075 (11)
C4	0.0480 (14)	0.0472 (13)	0.0444 (13)	0.0039 (11)	0.0150 (11)	−0.0043 (11)
C5	0.0430 (14)	0.0664 (16)	0.0576 (16)	0.0005 (12)	0.0108 (12)	−0.0223 (13)
C6	0.0514 (16)	0.0545 (15)	0.0722 (18)	−0.0128 (12)	0.0285 (14)	−0.0229 (14)
O4	0.0520 (10)	0.0442 (9)	0.0527 (10)	0.0011 (7)	0.0203 (8)	−0.0131 (7)
C41	0.0446 (13)	0.0415 (12)	0.0521 (14)	0.0040 (10)	0.0076 (11)	−0.0054 (11)
C141	0.0513 (14)	0.0397 (12)	0.0453 (13)	0.0080 (11)	0.0082 (11)	−0.0019 (10)
C142	0.0510 (15)	0.0579 (15)	0.0441 (14)	0.0011 (12)	0.0058 (12)	−0.0045 (12)
C143	0.0581 (17)	0.0772 (19)	0.0501 (15)	0.0059 (14)	0.0130 (13)	0.0065 (14)
C144	0.088 (2)	0.0690 (18)	0.0540 (17)	0.0236 (17)	0.0277 (16)	0.0054 (15)
C145	0.122 (3)	0.0537 (17)	0.070 (2)	0.0072 (18)	0.040 (2)	−0.0174 (15)
C146	0.100 (2)	0.0518 (16)	0.0696 (19)	−0.0091 (15)	0.0334 (18)	−0.0129 (14)
C151	0.0498 (14)	0.0416 (12)	0.0521 (14)	0.0045 (11)	0.0171 (12)	−0.0075 (11)
C152	0.0595 (17)	0.0526 (15)	0.0755 (19)	0.0007 (13)	0.0234 (15)	−0.0081 (14)
C153	0.079 (2)	0.0557 (17)	0.106 (3)	0.0001 (16)	0.047 (2)	−0.0010 (18)
C154	0.117 (3)	0.0544 (17)	0.083 (2)	0.0105 (19)	0.056 (2)	0.0096 (16)
C155	0.092 (2)	0.0709 (19)	0.0646 (19)	0.0251 (18)	0.0133 (17)	0.0088 (16)
C156	0.0614 (17)	0.0567 (16)	0.0640 (17)	0.0072 (13)	0.0131 (14)	0.0013 (14)
C11	0.109 (3)	0.0547 (16)	0.110 (3)	0.0158 (17)	0.069 (2)	0.0279 (17)
C12	0.089 (2)	0.0500 (15)	0.086 (2)	0.0124 (15)	0.0381 (18)	0.0181 (15)
C13	0.087 (2)	0.0635 (18)	0.095 (2)	0.0207 (16)	−0.0026 (19)	−0.0269 (17)
C14	0.073 (2)	0.0543 (16)	0.083 (2)	0.0038 (15)	0.0015 (17)	−0.0062 (15)
O14	0.0782 (17)	0.0650 (14)	0.200 (3)	−0.0039 (12)	−0.0078 (17)	−0.0152 (16)
C161	0.0586 (17)	0.0523 (15)	0.085 (2)	0.0033 (14)	0.0038 (15)	−0.0064 (14)
C162	0.071 (3)	0.063 (2)	0.125 (5)	0.028 (2)	−0.031 (3)	−0.023 (3)
C163	0.062 (2)	0.065 (2)	0.136 (5)	0.0107 (19)	−0.023 (3)	−0.029 (3)
C164	0.0531 (16)	0.0537 (15)	0.0714 (18)	0.0057 (13)	0.0157 (14)	−0.0063 (13)
C165	0.064 (2)	0.056 (2)	0.181 (11)	0.0166 (17)	−0.013 (3)	−0.014 (4)
C166	0.061 (2)	0.057 (2)	0.170 (10)	0.0066 (17)	−0.017 (3)	−0.011 (3)
C171	0.0586 (17)	0.0523 (15)	0.085 (2)	0.0033 (14)	0.0038 (15)	−0.0064 (14)
C172	0.071 (3)	0.063 (2)	0.125 (5)	0.028 (2)	−0.031 (3)	−0.023 (3)
C173	0.062 (2)	0.065 (2)	0.136 (5)	0.0107 (19)	−0.023 (3)	−0.029 (3)
C174	0.0531 (16)	0.0537 (15)	0.0714 (18)	0.0057 (13)	0.0157 (14)	−0.0063 (13)
C175	0.064 (2)	0.056 (2)	0.181 (11)	0.0166 (17)	−0.013 (3)	−0.014 (4)
C176	0.061 (2)	0.057 (2)	0.170 (10)	0.0066 (17)	−0.017 (3)	−0.011 (3)
C181	0.0610 (17)	0.0529 (15)	0.0659 (17)	0.0014 (13)	0.0175 (14)	0.0020 (13)
C182	0.121 (3)	0.087 (2)	0.073 (2)	−0.007 (2)	0.028 (2)	0.0090 (18)
C183	0.079 (2)	0.069 (2)	0.124 (3)	0.0164 (17)	0.019 (2)	−0.014 (2)
C184	0.085 (2)	0.0685 (18)	0.074 (2)	−0.0021 (16)	0.0180 (17)	−0.0076 (16)
C21	0.0455 (13)	0.0423 (12)	0.0432 (13)	−0.0045 (10)	0.0127 (11)	−0.0105 (10)
C22	0.0518 (14)	0.0467 (13)	0.0406 (13)	−0.0047 (11)	0.0124 (11)	−0.0067 (10)
C23	0.0479 (14)	0.0481 (13)	0.0496 (14)	0.0050 (11)	0.0062 (12)	−0.0117 (11)
C24	0.0476 (14)	0.0601 (15)	0.0527 (15)	−0.0056 (12)	0.0173 (12)	−0.0186 (13)
C25	0.0535 (15)	0.0581 (15)	0.0428 (14)	−0.0097 (12)	0.0169 (12)	−0.0076 (12)
C26	0.0536 (15)	0.0480 (13)	0.0472 (14)	−0.0034 (11)	0.0106 (12)	−0.0072 (11)
C27	0.0486 (15)	0.0505 (14)	0.0548 (16)	−0.0031 (12)	0.0166 (12)	−0.0165 (13)
O21	0.0677 (13)	0.0620 (12)	0.0798 (13)	0.0171 (10)	0.0207 (10)	−0.0063 (10)
O22	0.0747 (13)	0.0646 (11)	0.0634 (12)	0.0063 (9)	0.0386 (10)	−0.0071 (10)
N23	0.0668 (16)	0.0624 (15)	0.0715 (16)	0.0143 (12)	0.0064 (14)	−0.0117 (13)
O23	0.1038 (18)	0.0849 (15)	0.0873 (16)	0.0259 (13)	0.0133 (14)	0.0247 (13)
O24	0.0920 (17)	0.1013 (17)	0.1138 (19)	0.0466 (14)	0.0293 (15)	−0.0112 (15)
N25	0.0849 (19)	0.0748 (17)	0.0557 (15)	−0.0129 (14)	0.0292 (15)	−0.0015 (13)
O25	0.0960 (18)	0.127 (2)	0.0806 (16)	−0.0088 (15)	0.0543 (14)	−0.0002 (14)
O26	0.132 (2)	0.1008 (18)	0.0844 (17)	0.0167 (16)	0.0413 (16)	0.0340 (15)

### Geometric parameters (Å, º)

**Table:**

N1—C2	1.491 (3)	C13—H13A	0.9700
N1—C11	1.493 (3)	C13—H13B	0.9700
N1—C6	1.496 (3)	C14—O14	1.201 (3)
N1—H1	0.99 (3)	C14—C161	1.484 (4)
C2—C3	1.503 (3)	C161—C166	1.344 (5)
C2—H2B	0.9700	C161—C162	1.397 (5)
C2—H2A	0.9700	C162—C163	1.382 (5)
C3—C4	1.517 (3)	C162—H162	0.9300
C3—H3A	0.9700	C163—C164	1.396 (4)
C3—H3B	0.9700	C163—H163	0.9300
C4—O4	1.434 (3)	C164—C165	1.355 (6)
C4—C5	1.507 (3)	C164—C181	1.528 (4)
C4—H4	0.9800	C165—C166	1.378 (5)
C5—C6	1.504 (4)	C165—H165	0.9300
C5—H5A	0.9700	C166—H166	0.9300
C5—H5B	0.9700	C172—C173	1.381 (8)
C6—H6A	0.9700	C172—H172	0.9300
C6—H6B	0.9700	C173—H173	0.9300
O4—C41	1.423 (3)	C175—C176	1.376 (8)
C41—C151	1.510 (3)	C175—H175	0.9300
C41—C141	1.526 (3)	C176—H176	0.9300
C41—H41	0.9800	C181—C183	1.517 (4)
C141—C142	1.377 (3)	C181—C182	1.526 (4)
C141—C146	1.386 (3)	C181—C184	1.530 (4)
C142—C143	1.390 (3)	C182—H18A	0.9600
C142—H142	0.9300	C182—H18B	0.9600
C143—C144	1.368 (4)	C182—H18C	0.9600
C143—H143	0.9300	C183—H18D	0.9600
C144—C145	1.368 (4)	C183—H18E	0.9600
C144—H144	0.9300	C183—H18F	0.9600
C145—C146	1.384 (4)	C184—H18G	0.9600
C145—H145	0.9300	C184—H18H	0.9600
C146—H146	0.9300	C184—H18I	0.9600
C151—C152	1.382 (3)	C21—C26	1.380 (3)
C151—C156	1.383 (4)	C21—C22	1.383 (3)
C152—C153	1.379 (4)	C21—C27	1.524 (3)
C152—H152	0.9300	C22—C23	1.378 (3)
C153—C154	1.363 (5)	C22—H22	0.9300
C153—H153	0.9300	C23—C24	1.371 (3)
C154—C155	1.382 (5)	C23—N23	1.473 (3)
C154—H154	0.9300	C24—C25	1.370 (3)
C155—C156	1.381 (4)	C24—H24	0.9300
C155—H155	0.9300	C25—C26	1.381 (3)
C156—H156	0.9300	C25—N25	1.472 (3)
C11—C12	1.512 (4)	C26—H26	0.9300
C11—H11A	0.9700	C27—O21	1.226 (3)
C11—H11B	0.9700	C27—O22	1.265 (3)
C12—C13	1.481 (4)	N23—O24	1.215 (3)
C12—H12A	1.0216	N23—O23	1.215 (3)
C12—H12B	0.8703	N25—O26	1.215 (3)
C13—C14	1.501 (4)	N25—O25	1.215 (3)
			
C2—N1—C11	112.0 (2)	C11—C12—H12A	113.2
C2—N1—C6	110.00 (18)	C13—C12—H12B	107.6
C11—N1—C6	110.5 (2)	C11—C12—H12B	110.1
C2—N1—H1	107.3 (14)	H12A—C12—H12B	110.5
C11—N1—H1	109.0 (14)	C12—C13—C14	116.4 (3)
C6—N1—H1	107.9 (15)	C12—C13—H13A	108.2
N1—C2—C3	111.04 (19)	C14—C13—H13A	108.2
N1—C2—H2B	109.4	C12—C13—H13B	108.2
C3—C2—H2B	109.4	C14—C13—H13B	108.2
N1—C2—H2A	109.4	H13A—C13—H13B	107.3
C3—C2—H2A	109.4	O14—C14—C161	120.9 (3)
H2B—C2—H2A	108.0	O14—C14—C13	120.9 (3)
C2—C3—C4	111.94 (19)	C161—C14—C13	118.2 (3)
C2—C3—H3A	109.2	C166—C161—C162	117.5 (3)
C4—C3—H3A	109.2	C166—C161—C14	121.8 (3)
C2—C3—H3B	109.2	C162—C161—C14	120.3 (3)
C4—C3—H3B	109.2	C163—C162—C161	120.0 (4)
H3A—C3—H3B	107.9	C163—C162—H162	120.0
O4—C4—C5	105.72 (18)	C161—C162—H162	120.0
O4—C4—C3	113.48 (19)	C162—C163—C164	122.1 (4)
C5—C4—C3	108.75 (19)	C162—C163—H163	119.0
O4—C4—H4	109.6	C164—C163—H163	119.0
C5—C4—H4	109.6	C165—C164—C163	115.2 (3)
C3—C4—H4	109.6	C165—C164—C181	124.3 (3)
C6—C5—C4	111.3 (2)	C163—C164—C181	120.2 (3)
C6—C5—H5A	109.4	C164—C165—C166	123.3 (5)
C4—C5—H5A	109.4	C164—C165—H165	118.3
C6—C5—H5B	109.4	C166—C165—H165	118.3
C4—C5—H5B	109.4	C161—C166—C165	121.3 (5)
H5A—C5—H5B	108.0	C161—C166—H166	119.3
N1—C6—C5	111.49 (19)	C165—C166—H166	119.3
N1—C6—H6A	109.3	C173—C172—H172	119.0
C5—C6—H6A	109.3	C172—C173—H173	120.1
N1—C6—H6B	109.3	C176—C175—H175	118.8
C5—C6—H6B	109.3	C175—C176—H176	118.7
H6A—C6—H6B	108.0	C183—C181—C182	109.0 (3)
C41—O4—C4	116.44 (17)	C183—C181—C164	112.3 (2)
O4—C41—C151	112.52 (19)	C182—C181—C164	109.4 (2)
O4—C41—C141	106.93 (18)	C183—C181—C184	107.5 (2)
C151—C41—C141	112.66 (18)	C182—C181—C184	108.4 (2)
O4—C41—H41	108.2	C164—C181—C184	110.1 (2)
C151—C41—H41	108.2	C181—C182—H18A	109.5
C141—C41—H41	108.2	C181—C182—H18B	109.5
C142—C141—C146	117.8 (2)	H18A—C182—H18B	109.5
C142—C141—C41	122.5 (2)	C181—C182—H18C	109.5
C146—C141—C41	119.7 (2)	H18A—C182—H18C	109.5
C141—C142—C143	121.1 (2)	H18B—C182—H18C	109.5
C141—C142—H142	119.4	C181—C183—H18D	109.5
C143—C142—H142	119.4	C181—C183—H18E	109.5
C144—C143—C142	120.1 (3)	H18D—C183—H18E	109.5
C144—C143—H143	120.0	C181—C183—H18F	109.5
C142—C143—H143	120.0	H18D—C183—H18F	109.5
C145—C144—C143	119.7 (3)	H18E—C183—H18F	109.5
C145—C144—H144	120.2	C181—C184—H18G	109.5
C143—C144—H144	120.2	C181—C184—H18H	109.5
C144—C145—C146	120.3 (3)	H18G—C184—H18H	109.5
C144—C145—H145	119.9	C181—C184—H18I	109.5
C146—C145—H145	119.9	H18G—C184—H18I	109.5
C145—C146—C141	121.0 (3)	H18H—C184—H18I	109.5
C145—C146—H146	119.5	C26—C21—C22	119.2 (2)
C141—C146—H146	119.5	C26—C21—C27	120.1 (2)
C152—C151—C156	118.5 (2)	C22—C21—C27	120.7 (2)
C152—C151—C41	120.3 (2)	C23—C22—C21	119.6 (2)
C156—C151—C41	121.2 (2)	C23—C22—H22	120.2
C153—C152—C151	121.4 (3)	C21—C22—H22	120.2
C153—C152—H152	119.3	C24—C23—C22	122.4 (2)
C151—C152—H152	119.3	C24—C23—N23	118.5 (2)
C154—C153—C152	119.7 (3)	C22—C23—N23	119.2 (2)
C154—C153—H153	120.2	C25—C24—C23	117.0 (2)
C152—C153—H153	120.2	C25—C24—H24	121.5
C153—C154—C155	120.0 (3)	C23—C24—H24	121.5
C153—C154—H154	120.0	C24—C25—C26	122.6 (2)
C155—C154—H154	120.0	C24—C25—N25	117.6 (2)
C156—C155—C154	120.4 (3)	C26—C25—N25	119.8 (2)
C156—C155—H155	119.8	C21—C26—C25	119.3 (2)
C154—C155—H155	119.8	C21—C26—H26	120.3
C155—C156—C151	120.1 (3)	C25—C26—H26	120.3
C155—C156—H156	120.0	O21—C27—O22	127.0 (2)
C151—C156—H156	120.0	O21—C27—C21	118.1 (2)
N1—C11—C12	114.0 (2)	O22—C27—C21	115.0 (2)
N1—C11—H11A	108.8	O24—N23—O23	124.5 (3)
C12—C11—H11A	108.8	O24—N23—C23	117.9 (3)
N1—C11—H11B	108.8	O23—N23—C23	117.7 (2)
C12—C11—H11B	108.8	O26—N25—O25	124.2 (3)
H11A—C11—H11B	107.7	O26—N25—C25	117.6 (3)
C13—C12—C11	111.0 (3)	O25—N25—C25	118.1 (3)
C13—C12—H12A	104.2		
			
C11—N1—C2—C3	−179.77 (19)	O14—C14—C161—C166	10.3 (6)
C6—N1—C2—C3	−56.5 (2)	C13—C14—C161—C166	−170.9 (5)
N1—C2—C3—C4	57.2 (2)	O14—C14—C161—C162	−162.5 (4)
C2—C3—C4—O4	61.4 (2)	C13—C14—C161—C162	16.4 (5)
C2—C3—C4—C5	−55.9 (2)	C166—C161—C162—C163	5.6 (7)
O4—C4—C5—C6	−66.3 (2)	C14—C161—C162—C163	178.6 (4)
C3—C4—C5—C6	55.8 (3)	C161—C162—C163—C164	0.8 (7)
C2—N1—C6—C5	57.0 (2)	C162—C163—C164—C165	−6.2 (8)
C11—N1—C6—C5	−178.8 (2)	C162—C163—C164—C181	−179.9 (4)
C4—C5—C6—N1	−57.7 (3)	C163—C164—C165—C166	5.7 (11)
C5—C4—O4—C41	−179.83 (19)	C181—C164—C165—C166	179.0 (6)
C3—C4—O4—C41	61.1 (3)	C162—C161—C166—C165	−6.3 (10)
C4—O4—C41—C151	53.7 (3)	C14—C161—C166—C165	−179.2 (6)
C4—O4—C41—C141	177.88 (18)	C164—C165—C166—C161	0.5 (13)
O4—C41—C141—C142	4.6 (3)	C165—C164—C181—C183	−24.9 (6)
C151—C41—C141—C142	128.7 (2)	C163—C164—C181—C183	148.2 (4)
O4—C41—C141—C146	−176.5 (2)	C165—C164—C181—C182	96.3 (6)
C151—C41—C141—C146	−52.4 (3)	C163—C164—C181—C182	−90.6 (4)
C146—C141—C142—C143	1.4 (4)	C165—C164—C181—C184	−144.6 (6)
C41—C141—C142—C143	−179.7 (2)	C163—C164—C181—C184	28.4 (4)
C141—C142—C143—C144	−0.2 (4)	C26—C21—C22—C23	1.4 (3)
C142—C143—C144—C145	−1.2 (4)	C27—C21—C22—C23	−176.3 (2)
C143—C144—C145—C146	1.4 (5)	C21—C22—C23—C24	−0.9 (4)
C144—C145—C146—C141	−0.1 (5)	C21—C22—C23—N23	178.1 (2)
C142—C141—C146—C145	−1.3 (4)	C22—C23—C24—C25	0.0 (4)
C41—C141—C146—C145	179.8 (3)	N23—C23—C24—C25	−179.0 (2)
O4—C41—C151—C152	−129.7 (2)	C23—C24—C25—C26	0.4 (4)
C141—C41—C151—C152	109.3 (2)	C23—C24—C25—N25	−179.6 (2)
O4—C41—C151—C156	50.5 (3)	C22—C21—C26—C25	−1.0 (3)
C141—C41—C151—C156	−70.5 (3)	C27—C21—C26—C25	176.7 (2)
C156—C151—C152—C153	−0.6 (4)	C24—C25—C26—C21	0.1 (4)
C41—C151—C152—C153	179.6 (2)	N25—C25—C26—C21	−180.0 (2)
C151—C152—C153—C154	−0.2 (4)	C26—C21—C27—O21	1.3 (3)
C152—C153—C154—C155	0.6 (5)	C22—C21—C27—O21	179.0 (2)
C153—C154—C155—C156	−0.3 (5)	C26—C21—C27—O22	−177.2 (2)
C154—C155—C156—C151	−0.5 (4)	C22—C21—C27—O22	0.5 (3)
C152—C151—C156—C155	0.9 (4)	C24—C23—N23—O24	−4.2 (4)
C41—C151—C156—C155	−179.3 (2)	C22—C23—N23—O24	176.8 (2)
C2—N1—C11—C12	−72.4 (3)	C24—C23—N23—O23	174.9 (2)
C6—N1—C11—C12	164.5 (3)	C22—C23—N23—O23	−4.1 (4)
N1—C11—C12—C13	−122.9 (3)	C24—C25—N25—O26	168.7 (3)
C11—C12—C13—C14	−179.2 (3)	C26—C25—N25—O26	−11.3 (4)
C12—C13—C14—O14	−1.7 (5)	C24—C25—N25—O25	−9.9 (4)
C12—C13—C14—C161	179.5 (3)	C26—C25—N25—O25	170.2 (2)

### Hydrogen-bond geometry (Å, º)

**Table:**

*D*—H···*A*	*D*—H	H···*A*	*D*···*A*	*D*—H···*A*
N1—H1···O22	0.99 (3)	1.66 (2)	2.634 (3)	167 (2)
C2—H2*A*···O25^i^	0.97	2.50	3.444 (3)	163
C11—H11*A*···O14^ii^	0.97	2.49	3.358 (4)	150

Symmetry codes: (i) *x*−1, −*y*+3/2, *z*+1/2; (ii) *x*−1, *y*, *z*.
